# Y‐chromosome haplotypes are associated with variation in size and age at maturity in male Chinook salmon

**DOI:** 10.1111/eva.13084

**Published:** 2020-08-28

**Authors:** Garrett J. McKinney, James E. Seeb, Carita E. Pascal, Daniel E. Schindler, Sara E. Gilk‐Baumer, Lisa W. Seeb

**Affiliations:** ^1^ School of Aquatic and Fishery Sciences University of Washington Seattle WA USA; ^2^ Alaska Department of Fish and Game Anchorage AK USA

**Keywords:** age at maturity, Chinook salmon, GT‐seq, haplotype, RADseq, size at maturity, Ychromosome

## Abstract

Variation in size and age at maturity is an important component of life history that is influenced by both environmental and genetic factors. In salmonids, large size confers a direct reproductive advantage through increased fecundity and egg quality in females, while larger males gain a reproductive advantage by monopolizing access to females. In addition, variation in size and age at maturity in males can be associated with different reproductive strategies; younger smaller males may gain reproductive success by sneaking among mating pairs. In both sexes, there is a trade‐off between older age and increased reproductive success and increased risk of mortality by delaying reproduction. We identified four Y‐chromosome haplogroups that showed regional‐ and population‐specific variation in frequency using RADseq data for 21 populations of Alaska Chinook salmon. We then characterized the range‐wide distribution of these haplogroups using GT‐seq assays. These haplogroups exhibited associations with size at maturity in multiple populations, suggesting that lack of recombination between X and Y‐chromosomes has allowed Y‐chromosome haplogroups to capture different alleles that influence size at maturity. Ultimately, conservation of life history diversity in Chinook salmon may require conservation of Y‐chromosome haplotype diversity.

## 1 INTRODUCTION

Variation in life history within populations is common across taxa and is often associated with alternative strategies for increasing fitness. This includes partial migration, where some individuals of a population migrate while others remain resident (Chapman, Brönmark, Nilsson, & Hansson, 2011), reproductive morphs that exhibit different mating strategies or sexually selected traits (Johnston et al., 2013; Küpper et al., 2015; Shuster, 1989), age and size at maturity (Gibbons, Semlitsch, Greene, & Schubauer, 1981), and even length of life span such as annual versus perennial plants (Hall, Basten, & Willis, 2006). While life history variation is often assumed to be under the influence of many genes of small effect, examples of a large‐effect genes and supergenes (sets of linked genes) influencing life history variation are increasingly being found. These include single genes that influence age at maturity (Barson et al., 2015) and sexually selected traits (Johnston et al., 2013); these also include chromosome inversions contributing to annual versus perennial life history (Twyford & Friedman, 2015) and variation in migration versus residency (Pearse, Miller, Abadia‐Cardoso, & Garza, 2014). The mechanisms underlying variation in life history have important implications for how this important diversity is maintained under different selective regimes.

Variation in life history strategies is exhibited by many salmon species with size and age at maturity being an important component of this variation. In females, large body size confers a direct reproductive advantage through increased fecundity and egg quality (Healey & Heard, 1984). In contrast, variation in size and age at maturity in males can be associated with different reproductive strategies that exhibit frequency‐dependent fitness. For example, older larger males gain reproductive success by monopolizing access to females, while younger smaller males gain reproductive success by sneaking in among mating pairs (Berejikian et al., 2010; Healey & Heard, 1984). For both sexes, there is a trade‐off to delayed maturation where increased reproductive success is countered by an increased risk of mortality before reproduction. The optimal age at maturity for a population should represent the balance between reproductive benefits and mortality costs of delayed maturation (Healey, 1986), and forces that change these costs or benefits could result in shifts in age composition. Nonetheless, considerable diversity in age at maturation is maintained within many populations, presumably as a bet‐hedging strategy to spread risks over the life cycle of these fish.

Age at maturity in salmon is generally thought to be a threshold trait that is dependent either upon reaching a minimum size at age or upon growth rate at key periods (Healey, 1991; Thorpe, 2007). Environmental factors that influence growth rate have been shown to influence age at maturity in many species. In the wild, studies show correlations between ocean conditions such as temperature (or productivity) and patterns of age at maturity (Otero et al., 2012; Siegel, McPhee, & Adkison, 2017). In experimental settings, age at maturity was manipulated through changing temperature (Harstad et al., 2018; Heath, Devlin, Heath, & Iwama, 1994) or food ration (Larsen et al., 2006; Rowe & Thorpe, 1990).

In addition to environmental effects, multiple lines of evidence show a genetic component to age at maturity. High heritability values suggest considerable genetic variation for age at maturity in several salmon species (Gall, Baltodano, & Huang, 1988; Gjerde, 1984; Hankin, Nicholas, & Downey, 1993; Heath et al., 1994), and quantitative trait locus (QTL) and genome‐wide association (GWAS) studies identified genomic regions associated with variation in age at maturity (Barson et al., 2015; Kodama, Hard, & Naish, 2018; Micheletti & Narum, 2018; Waters et al., 2018). Studies also demonstrated that offspring of alternative male phenotypes exhibit different growth rates (Berejikian, Van Doornik, & Atkins, 2011; Garant, Fontaine, Good, Dodson, & Bernatchez, 2002) with offspring of early maturation phenotypes (grilse and jacks) exhibiting high growth rates. In Atlantic salmon (*Salmo salar*), individuals with different life histories exhibit differing maturation thresholds that are genetically based (Aubin Horth & Dodson, 2004). Despite these findings, the genetic basis of maturation age in most salmonids remains poorly understood.

One complicating factor is that the genetic basis underlying variation in age at maturity appears to vary among salmonid species and even among populations within a species. In Atlantic salmon, age at maturity is strongly influenced by a single gene (VGLL3) (Ayllon et al., 2015; Barson et al., 2015). This gene exhibits sex‐dependent dominance, which facilitates sexually antagonistic selection (Barson et al., 2015). While this gene explained 39% of the phenotypic variability in European Atlantic salmon, studies in North American Atlantic salmon have shown that this association varies by population (Boulding, Ang, Elliott, Powell, & Schaeffer, 2019; Kusche et al., 2017) and this gene has not been shown to have an effect in Pacific salmon (genus *Oncorhynchus)* (Micheletti & Narum, 2018).

Chinook salmon (*O. tshawytscha*) are the largest of the Pacific salmon and follow various life history strategies, spending 0 to 2 years in fresh water and 1 to 4 or more years in the ocean (Riddell et al., 2018). Male Chinook salmon exhibit significant variation in size and age at maturity (Healey, 1991) that is linked to differential reproductive tactics and is likely controlled by both environmental and genetic components (Berejikian et al., 2010; Young, Conti, & Dean, 2013). Many populations throughout North America recently experienced marked declines in size and age at maturity, which may erode life history variation (Lewis, Grant, Brenner, & Hamazaki, 2015; Ohlberger, Ward, Schindler, & Lewis, 2018). Explanations for decreased age at maturity have focused on the impacts of fisheries‐induced evolution (Hard et al., 2008; Kendall, Dieckmann, Heino, Punt, & Quinn, 2014) or changing environmental conditions (Siegel et al., 2017); however, these factors alone are not consistent nor sufficient to explain current declines, and it is likely that these declines are driven by multiple complex factors (Ohlberger et al., 2018). While the genetic control of age at maturity in Chinook salmon is still poorly understood, past studies offer clues to genomic regions that may be associated with maturation age. In particular, Heath, Rankin, Bryden, Heath, and Shrimpton (2002) identified a strong sex‐linked component to age at maturity in Chinook salmon, suggesting the influence of genes on the Y chromosome.

The X and Y chromosomes in most salmonid species are morphologically undifferentiated (Davidson, Huang, Fujiki, von Schalburg, & Koop, 2009). Available sequence data suggest that the primary difference between sex chromosomes is an insertion containing the sex‐determining gene (SDY, Yano et al., 2013), and in Chinook salmon, a 2.4 Mb male‐specific repetitive sequence has been identified (Devlin, Stone, & Smailus, 1998). While the sex‐determining gene has been assigned to chromosome 17 (Ots17) in Chinook salmon (Phillips, Park, & Naish, 2013), the region that contains this gene is not in either of the current genome assemblies (Christensen et al., 2018; Narum, Genova, Micheletti, & Maass, 2018), so the exact location of *sdY* is unknown. The genome assembly by Christensen et al. (2018) was of a female, while the assembly by Narum et al. (2018) used a male Chinook salmon; however, *sdY* was not assembled as part of a chromosome, likely because of extended repetitive sequence in this region (cf., Devlin et al., 1998).

Despite a lack of large‐scale differentiation, the X‐ and Y‐chromosomes could show sequence divergence due to sex‐specific patterns of recombination (heterochiasmy, see Sardell & Kirkpatrick, 2020). Recombination in females takes place along the full length of the chromosome, while recombination in males is strongly localized to telomeric regions (Lien et al., 2011; Sakamoto et al., 2000), restricting recombination between the X‐ and Y‐chromosomes. Reduced recombination between sex chromosomes is common across taxa, and in salmonids is supported by a 33 Mb signal of sex association observed in Atlantic salmon (Kijas et al., 2018) and a lack of recombination between the sex‐determining region and an allozyme locus on the sex chromosome in Chinook salmon (Marshall, Knudsen, & Allendorf, 2004). In addition to facilitating divergence between sex chromosomes, sex‐limited recombination could lead to the formation of different Y‐chromosome haplotypes and the capture of adaptive genetic variants (Bergero, Gardner, Bader, Yong, & Charlesworth, 2019). If Y‐chromosome haplotypes have sufficiently diverged from the X‐chromosome, they may be identified through patterns of extended linkage disequilibrium.

We examined patterns of linkage disequilibrium on the sex chromosome of Chinook salmon to determine whether male‐specific haplotype blocks (Y‐chromosome haplotypes) existed, and if so, are these haplotypes associated with variation in size and age at maturity which commonly differ between sexes in Chinook salmon. We identified four Y‐chromosome haplogroups (groups of similar haplotypes) in Chinook salmon from Alaska that showed regional‐ and population‐specific variation in frequency. These haplogroups showed associations with size at maturity in multiple populations, suggesting that the lack of recombination between X‐ and Y‐chromosomes has allowed genetic variants influencing size and age at maturity to segregate on different Y‐chromosome haplogroups.

## 2 MATERIALS AND METHODS

### 2.1 RAD Y‐chromosome haplotypes

**FIGURE 1:**
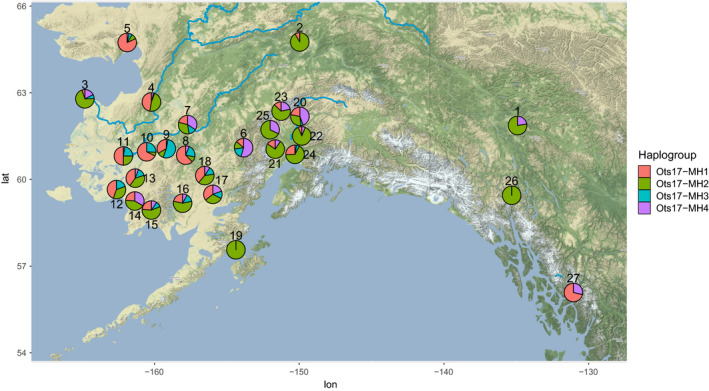
Frequency of Y‐chromosome haplogroups throughout Alaska based on RADseq and GT‐seq data. Locations of populations are approximate to prevent overlap of pie charts. Population names are given in Table 1. Note that the location for the Lower Yukon Test Fishery (population 3) indicates where fish were caught on their return to the Yukon River; these samples may represent fish from many populations that spawn throughout the Yukon River

We used existing RADseq data to examine patterns of genetic variation in the sex chromosome. RADseq data for 21 populations of Chinook salmon from Alaska were obtained from: NCBI SRA accessions SRP034950 (Larson, Seeb, Pascal, Templin, & Seeb, 2014) and SRP129894 (McKinney, Waples, Pascal, Seeb, & Seeb, 2018), bioproject PRJNA560365 (McKinney, Pascal, et al., 2020), and raw data used in Dann et al. (2018). Raw data from Dann et al. (2018) are available from those authors upon request. Populations ranged from Cook Inlet to the Upper Yukon River and include a total of 1,082 samples (Figure 1, Table 1). RADseq data were processed with Stacks V1.7 (Catchen, Hohenlohe, Bassham, Amores, & Cresko, 2013; Catchen, Amores, Hohenlohe, Cresko, & Postlethwait, 2011) using default settings with the following exceptions: process_radtags (‐c ‐r ‐q ‐filter_illumina ‐t 94), ustacks (‐m 2 ‐M 2, ‐‐model_type bounded ‐‐bound_high 0.05), cstacks (‐n 2). The Stacks catalog from McKinney, Pascal, et al. (2020) was used for genotyping to keep consistent RADtag names among this and previous studies. A total of six individuals per population from Cook Inlet were added to this catalog to allow for additional allelic variation.

**TABLE 1:** Populations used in this study (Lower Yukon Test Fishery is a mixture of populations). RADseq data were used for haplogroup discovery, while GT‐seq data were used to confirm that haplogroups were male‐specific and to expand the geographic distribution of haplogroups

Map number	Population	Data type	Region	Latitude	Longitude	*N*	Ots17‐MH1	Ots17‐MH2	Ots17‐MH3	Ots17‐MH4	*N* aged males
A											
1	Big Salmon River	RAD	Yukon River^a^	61.87	−134.92	49	0.00	0.78	0.00	0.22	0
2	Kantishna River	GT‐seq	Yukon River	64.76	−149.97	48	0.09	0.91	0.00	0.00	0
3	Lower Yukon Test Fishery	GT‐seq	Yukon River	62.79	−164.81	95	0.05	0.70	0.07	0.18	57
4	Anvik River	RAD	Yukon River	62.68	−160.21	56	0.47	0.47	0.02	0.04	0
5	Tubutulik River	RAD	Western Alaska	64.74	−161.89	56	0.82	0.09	0.06	0.03	0
6	Necons River	RAD	Western Alaska	61.10	−153.85	47	0.14	0.14	0.18	0.55	0
7	George River	RAD/GT‐seq	Western Alaska	61.90	−157.71	48/47	0.21	0.32	0.13	0.34	0
8	Kogrukluk River	RAD	Western Alaska	60.84	−157.85	59	0.63	0.09	0.22	0.06	0
9	Aniak River	RAD	Western Alaska	61.06	−159.18	47	0.33	0.11	0.53	0.03	0
10	Kisaralik River	RAD	Western Alaska	60.86	−161.24	48	0.68	0.06	0.26	0.00	0
11	Kwethluk River	RAD	Western Alaska	60.81	−161.45	47	0.50	0.25	0.22	0.03	0
12	Kanektok River	RAD/GT‐seq	Western Alaska	59.75	−161.93	48/47	0.44	0.36	0.19	0.00	0
13	Arolik River	RAD	Western Alaska	59.69	−161.88	48	0.40	0.40	0.14	0.06	0
14	Goodnews River	RAD	Western Alaska	59.25	−161.36	47	0.24	0.43	0.00	0.33	0
15	Togiak River	RAD	Western Alaska	59.09	−160.37	48	0.24	0.57	0.10	0.10	21
16	Iowithla River	RAD	Western Alaska	59.18	−158.06	48	0.22	0.57	0.14	0.08	0
17	Stuyahok River	RAD	Western Alaska	59.68	−156.17	48	0.35	0.32	0.13	0.19	0
18	Koktuli River	RAD	Western Alaska	59.94	−156.43	56	0.39	0.39	0.14	0.09	0
19	Karluk River	GT‐seq	Western Alaska	57.57	−154.38	48	0.00	1.00	0.00	0.00	25
20	Montana Creek	GT‐seq	Cook Inlet	62.18	−149.95	48	0.22	0.30	0.04	0.44	0
21	Chuitna River	RAD	Cook Inlet	61.20	−151.66	57	0.16	0.75	0.00	0.09	39
22	Sucker Creek	RAD	Cook Inlet	61.51	−150.83	57	0.06	0.88	0.00	0.06	0
23	Talachulitna River	RAD	Cook Inlet	61.62	−151.15	57	0.13	0.65	0.00	0.22	23
24	Theodore Creek	RAD	Cook Inlet	61.49	−151.09	56	0.26	0.67	0.05	0.03	0
25	Coal Creek	RAD	Cook Inlet	61.62	−151.76	55	0.00	0.68	0.00	0.32	19
26	Pullen Creek^b^	GT‐seq	Southeast Alaska	59.45	−135.32	48	0.00	1.00	0.00	0.00	0
27	Little Port Walter–Unuk River Stock^c^	GT‐seq	Southeast Alaska	56.09	−131.06	48	0.71	0.00	0.00	0.29	0
B											
–	Harrison River	GT‐seq	British Columbia	49.28	−121.92	48	NA	NA	NA	NA	0
–	Big Qualicum Hatchery	GT‐seq	British Columbia	49.40	−124.62	48	NA	NA	NA	NA	0
–	Kitwanga River	GT‐seq	British Columbia	55.10	−128.09	48	4	NA	NA	1	0
–	Kitsumkalum River	GT‐seq	British Columbia	54.52	−128.66	48	3	NA	NA	3	0
–	Morice River	GT‐seq	British Columbia	54.41	−126.75	48	1	NA	NA	6	0
–	Rapid River Hatchery	GT‐seq	Idaho	45.35	−116.40	48	NA	NA	NA	NA	0
–	McCall Fish Hatchery South Fork Salmon River	GT‐seq	Idaho	44.67	−115.71	48	NA	NA	NA	5	0
–	Marblemount Fish Hatchery	GT‐seq	Washington	48.52	−121.42	48	NA	NA	NA	NA	0
–	Soos Creek Hatchery	GT‐seq	Washington	47.31	−122.16	48	NA	NA	NA	NA	0
–	Columbia River at Wells Hatchery	GT‐seq	Washington	47.95	−119.87	48	NA	NA	NA	NA	0
–	Quinault Lake Pens	GT‐seq	Washington	47.47	−123.89	48	NA	NA	NA	NA	0
–	Lyons Ferry Hatchery	GT‐seq	Washington	46.59	−118.22	48	NA	NA	NA	2	0
–	Wenatchee River at Tumwater Dam	GT‐seq	Washington	47.61	−120.72	48	NA	NA	NA	7	0
–	Spring Creek Hatchery	GT‐seq	Washington	45.73	−121.55	48	NA	NA	NA	NA	0
–	Rock Creek, Umpqua River	GT‐seq	Oregon	43.34	−123.00	48	NA	NA	NA	NA	0
–	Cole River Hatchery, Rogue River	GT‐seq	Oregon	42.66	−122.69	48	NA	NA	NA	NA	0
–	Cedar Creek Hatchery	GT‐seq	Oregon	45.22	−123.84	48	NA	NA	NA	NA	0
–	McKenzie Hatchery	GT‐seq	Oregon	44.11	−122.68	48	NA	NA	NA	NA	0
–	Coleman National Fish Hatchery	GT‐seq	California	40.40	−122.14	48	NA	NA	NA	NA	0

Note

For populations with both RADseq and GT‐seq samples, samples sizes for RADseq are given first. Frequency of Y‐chromosome haplogroups is given for populations in Alaska in A. Due to the low proportion of males assigned to Y‐chromosome haplogroups outside of Alaska, the number of individuals assigned to each Y‐chromosome haplogroup for these populations is given in B.

^a^ The Yukon River is part of Western Alaska but was analyzed separately for this study.

^b^ Only one male from Pullen Creek was assigned to a Y‐chromosome haplogroup.

^c^ Only 11 out of 20 males from Little Port Walter–Unuk River Stock were assigned to a Y‐chromosome haplogroup.

RADseq results were filtered to remove SNPs that were likely to genotype poorly or be uninformative. These categories included collapsed paralogs, SNPs with more that 10% missing data, and SNPs with a minor allele frequency < 0.01. Paralogs are common in salmonid genomes due to an ancestral whole‐genome duplication (Allendorf & Thorgaard, 1984). With short‐read sequence, the two copies of paralog are often collapsed into a single locus that appears polyploid; these cannot be reliably genotyped in typical RADseq studies because of insufficient read depth (McKinney et al., 2018). Collapsed paralogs were identified using *HDplot* (McKinney, Waples, Seeb, & Seeb, 2017). Finally, loci were aligned to the Chinook salmon genome (Christensen et al., 2018) using Bowtie2 (Langmead & Salzberg, 2012) to determine genomic position; only loci that aligned to the sex chromosome (Ots17, Phillips et al., 2013) with fewer than four mismatches were retained for analysis.

Putative Y‐chromosome haplotypes were identified by examining patterns of linkage disequilibrium on Ots17 using network analysis. Genotypes for sets of high LD loci were phased into haplotypes representing the alleles on each chromosome pair within an individual and clustered based on similarity into haplogroups, which are groups of similar haplotypes. Network analysis of linkage disequilibrium is an effective approach for identifying genomic structures that exhibit high LD (Kemppainen et al., 2015) and has been used to identify distinct but overlapping haplotype blocks in chum salmon (McKinney, McPhee, Pascal, Seeb, & Seeb, 2020). Pairwise linkage disequilibrium was calculated using the *r* ^2^ method in *Plink* (V1.9) (Chang et al., 2015; Purcell et al., 2007), and SNP pairs with *r* ^2^ ≥ 0.3 were retained for network analysis. Network analysis was conducted using the igraph package in R (https://igraph.org/r) to identify sets of loci with high LD. Sets of loci that contained at least five SNPs and spanned at least 1 Mb were retained for further analysis. Genotypes for retained SNPs were then phased into haplotypes using fastPhase (Scheet & Stephens, 2006). Putative Y‐chromosome haplogroups were identified by clustering haplotypes using heatmap2 in R (Warnes et al., 2020) with the Ward.D clustering algorithm to minimize within‐group variance. Haplogroups that appear to be male‐specific are hereafter referred to as Y‐chromosome haplogroups and assigned names based on the following convention: chromosome number, MH for male haplogroup, followed by a sequential number, for example, Y‐chromosome haplogroups on chromosome 17 would be Ots17‐MH1, Ots17‐MH2, and so on. Allele frequencies within each haplogroup were visualized using logo plots with the ggseqlogo package in R (Wagih, 2017).

Assays for SNPs that were diagnostic for Y‐chromosome haplotypes were assembled into an amplicon panel (GT‐seq, Campbell, Harmon, & Narum, 2015) for expanded genotyping (see Methods in McKinney, Pascal, et al., 2020). Primers were designed using batch primer3 (You et al., 2008). Primer design used the consensus RAD sequence for each RADtag unless SNPs occurred within 20 bp of the end of the RADtag. In these cases, the consensus sequences were aligned to the genome using bowtie2 (Langmead & Salzberg, 2012), and genomic sequence flanking the RADtag was added to the consensus sequence for primer design. Default settings for SNP flanking primers were used with the following exceptions: a minimum amplicon size of 75 bp and a maximum amplicon size of 150 bp. Panel optimization followed the methods of McKinney, Pascal, et al. (2020); one round of sequencing using 80 individuals was conducted to identify loci that over‐amplified or produced unreliable genotypes. Amplification levels and genotyping accuracy were assessed using GT‐score (McKinney, Pascal, et al., 2020).

### 2.2 Expanded genotyping

Additional samples ranging from Alaska to California were genotyped using the GT‐seq panel to establish the geographic distribution of the haplogroups (Table 1). In addition to the Y‐chromosome haplogroup markers, this panel included the sex identification marker Ots_sexy3‐1 (Hess, Campbell, Matala, Hasselman, & Narum, 2016) to confirm that Y‐chromosome haplotypes were present only in male fish. A total of 1,341 samples from 27 populations were genotyped (Table 1); phenotypic sex was known for 193 of these. A total of 94 RADseq samples were included in the GT‐seq genotyping to examine concordance between the two datasets. Samples were sequenced on a HiSeq 4,000, and data were processed and genotyped using GT‐score (McKinney, Pascal, et al., 2020) available at https://github.com/gjmckinney/GTscore.

Two methods were used to assign GT‐seq samples to Y‐chromosome haplogroups. First, samples were assigned Y‐chromosome haplogroups using the same methods as the RADseq samples: phasing genotypes into haplotypes using fastPhase followed by haplotype clustering using heatmap2. Genotypes from RADseq samples were included in the GT‐seq haplotype assignment to assess concordance between the discriminatory ability of the full RADseq marker set and the subset that was successfully developed into GT‐seq markers. Second, the expected genotype patterns for males with each haplogroup were constructed assuming fixation of alleles on the X‐chromosome and the Y‐chromosome. The observed genotypes were then compared with the expected genotypes. Samples were assigned to a haplogroup if the observed genotypes had less than two mismatches to the expected genotypes.

### 2.3 Y‐chromosome haplotype analyses

Assignments were combined for GT‐seq and RADseq samples to characterize the distribution and frequency of Y‐chromosome haplogroups. Data for length and age at maturity were obtained from Alaska Department of Fish and Game (ADFG) for a subset of populations in Alaska and compared with Y‐chromosome haplogroup data to determine whether there were relationships between Y‐chromosome haplogroup and length and age at maturity. Analysis of variance (ANOVA) was used to test the significance of associations between Y‐chromosome haplogroups and length; population of origin was included as a covariate. A post hoc Tukey test was then performed to determine whether differences in size distribution were significant between individual haplogroups. The significance of associations between Y‐chromosome haplogroups and age at maturity of fish captured in the Lower Yukon River Test Fishery was assessed using an ANOVA with population of origin added as a covariate. There are multiple methods of reporting age in salmon (Koo, 1962); we report freshwater and ocean age for each individual using European notation, so an individual with an age of 1.3 would have spent 1 year growing in freshwater after emergence, followed by 3 years in the ocean, for a total age of 5 years.

## 3 RESULTS

A total of 448 SNPs from 323 RADtags remained after filtering SNPs with more than 10% missing data or a MAF ≤ 0.01, removing paralogs identified by HDplot, and retaining only SNPs that aligned to chromosome 17. Retained SNPs had an average coverage of 24x. When multiple SNPs occurred in the same RADtag, these were analyzed as independent SNPs.

### 3.1 RADseq Y‐haplotype discovery

**FIGURE 2:**
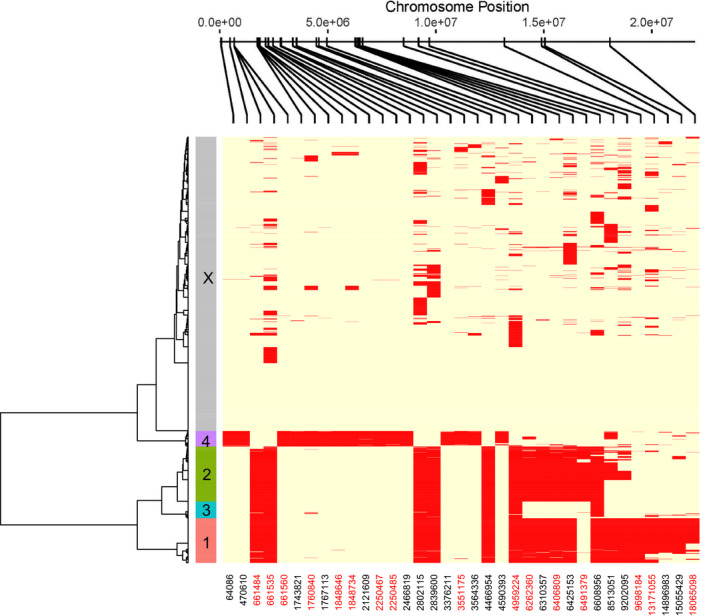
Plot of haplotype clusters identified by phasing high LD loci from the RADseq dataset. Each individual is represented by two haplotypes corresponding to each chromosome of Ots17. For each SNP, the most common allele is in yellow and the least common allele is in red. Haplotypes were clustered into haplogroups, and six major haplotype clusters were identified; these are denoted by different colors along the sample dendrogram (y‐axis). The gray haplogroup represents X‐chromosomes, while four haplogroups (pink = Ots17‐MH1, green = Ots17‐MH2, blue = Ots17‐MH3, and purple = Ots17‐MH4) represent Ychromosomes; numeric designations only are labeled on plot. SNP position on Ots17 is given on the x‐axis. The position for SNPs that were successfully developed into GT‐seq assays are color‐coded in red on the x‐axis. The relative position of each SNP on chromosome 17 is shown on the top of the x‐axis

Network analysis of linkage disequilibrium identified three sets of linked SNPs exhibiting long‐distance LD (4.5–9.5 Mb each). There were 35 SNPs in these high LD sets, representing 7.8% of the SNPs on Ots17 retained after filters. Individual genotypes for these SNPs were then phased, resulting in two haplotypes per individual. Clustering of haplotypes resulted in five major groupings that exhibited extended LD up to 17.4 Mb (Figure 2). Although phenotypic sex was available from only a subset of individuals, phased haplotypes from sexed individuals were present in each of these groupings (Table 2). One of the groupings contained phased haplotypes from both males and females, suggesting that this represents the X‐chromosome (gray cluster, Figure 2). Four of the haplogroups contained phased haplotypes predominantly from male samples (85%–95%), suggesting that these are from the Ychromosome (Table 2, Figure 2). In addition, all individuals with a haplotype in one of the Y‐chromosome haplogroups had their second haplotype in one of the X‐chromosome groupings.

**TABLE 2:** Number of haplotypes from RADseq samples assigned to each haplogroup by phenotypic sex

Heatmap group color	Sex			Putative chromosome	Haplogroup name
	F	M	Unknown		
Gray	168	114	999	X	X
Pink	1	21	206	Y	Ots17‐MH1
Green	9	55	54	Y	Ots17‐MH2
Blue	0	7	78	Y	Ots17‐MH3
Purple	2	11	66	Y	Ots17‐MH4

Note

Each individual has two haplotypes. Males should have one haplotype assigned to a Y‐chromosome haplogroup and one haplotype assigned to an X‐chromosome haplogroup. Females should have both haplotypes assigned to an X‐chromosome haplogroup.

All Y‐chromosome haplogroups were present, at varying frequency, in each geographic region, suggesting that these haplogroups are conserved throughout this broad geographic region (Figure 1, 1, 3and1, 3). Within Alaska, Y‐chromosome haplogroups showed regional variation in frequency with the Ots17‐MH1 haplotype exhibiting the high frequencies in most parts of Western Alaska and the Ots17‐MH4 haplotype the most frequent in Cook Inlet (Figure 1). While some females had haplotypes within the Y‐chromosome haplogroups, individuals had been visually sexed based on external features, which is known to have variable accuracy (Lozorie & McIntosh, 2014).

**TABLE 3:** Number of haplotypes from RADseq samples assigned to each haplogroup by region

Heatmap group color	Cook inlet	Western Alaska	Yukon River	Putative chromosome	Haplogroup name
Gray	410	942	139	X	X
Pink	20	183	25	Y	Ots17‐MH1
Green	112	130	39	Y	Ots17‐MH2
Blue	2	82	1	Y	Ots17‐MH3
Purple	20	53	6	Y	Ots17‐MH4

Note

Each individual has two haplotypes. Males should have one haplotype assigned to a Y‐chromosome haplogroup and one haplotype assigned to an X‐chromosome haplogroup. Females should have both haplotypes assigned to an X‐chromosome haplogroup.

**FIGURE 3:**
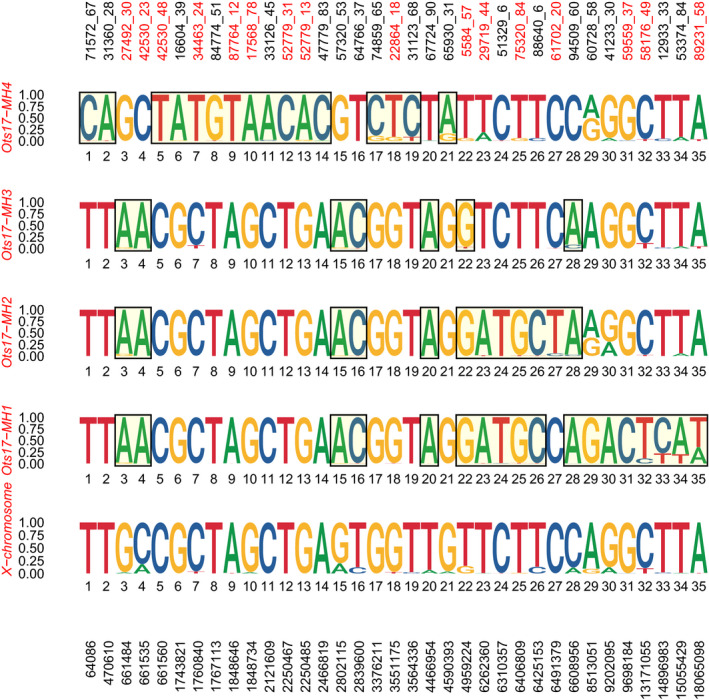
Logo plots showing allele frequencies within each of the male haplogroups and the X‐chromosome for the RADseq samples. For each SNP, the frequency of alleles within each haplotype (range = 0–1) is shown by the height of the allele. Alleles that are putatively fixed relative to the X‐chromosome are bounded by boxes. SNPs identified by tag number that were successfully converted to GT‐seq assays are in red on the top *x*‐axis. SNP positions on Ots17 are on the bottom *x*‐axis

Allele frequencies within each haplogroup were visualized using logo plots (Figure 3). A total of 35 SNPs were found to have fixed or nearly‐fixed differences in allele frequencies between Y‐chromosome haplogroups and the X‐chromosome. The Ots17‐MH1, Ots17‐MH2, and Ots17‐MH3 haplogroups shared seven of the SNPs that differentiated these haplogroups from the X‐chromosome (27492_30, 42530_30, 57320_53,64766_37,67724_90,5884_57,94509_60), suggesting a common evolutionary origin. The Ots17‐MH4 haplogroup shared no diagnostic SNPs with the other Y‐chromosome haplogroups. Several of the SNPs had allelic variation within the X‐chromosome (e.g., SNPs 57320_53 and 64766_37, Figure 3), suggesting that in some cases, existing polymorphisms on the X‐chromosome have become fixed for alternate alleles on different Y‐chromosome haplotypes. Alternatively, some SNPs exhibited no allelic variation within the X‐chromosome, suggesting that alternative alleles observed on the Y‐chromosome haplotypes may be novel genetic variants (e.g., SNP 71572_67; Figure 3).

### 3.2 GT‐seq Y‐chromosome expanded sampling

A GT‐seq panel was developed to genotype Y‐chromosome haplotype markers for a set of samples representing the North American range of Chinook salmon. A total of 23 of the 35 RAD SNPs passed filtering criteria prior to primer design; of these, 16 RAD SNPs were successfully converted to GT‐seq assays (Figure 3, Table S1). Samples genotyped with GT‐seq were assigned haplogroups using two methods, clustering of haplotypes using the Ward.D algorithm and assignment based on genotypes. Haplogroups Ots17‐MH1 and Ots17‐MH2 differed by several fixed SNPs in the RADseq dataset but only two fixed SNPs in the GT‐seq panel (Figure 3). For these haplogroups, an additional requirement was set that individuals must have the appropriate alleles for each of these fixed SNPs. For example, individuals in the Ots17‐MH2 haplogroup must have a T allele at position 6,491,379 and a G allele at position 9,698,184.

Samples that were genotyped with both RADseq and GT‐seq showed high concordance between RADseq and both GT‐seq haplogroup assignment methods except for Ots17‐MH3 (Table S2). When RADseq haplogroup assignment was compared with GT‐seq haplogroup assignment using Ward.D clustering, 10 of the 12 samples assigned to RADseq Ots17‐MH3 were grouped with females. This is likely because only three of the RADseq SNPs that differentiated this haplotype from the X‐chromosome were successfully developed into GT‐seq (Figure 3). In contrast, when GT‐seq samples were assigned to haplogroups based on comparing observed to expected genotypes for each haplogroup, 10 of the 12 samples correctly assigned to Ots17‐MH3, while two were not assigned to any haplogroup. For samples genotyped only with GT‐seq, there were no discrepancies in haplogroup assignment between the clustering and genotype‐based methods except for Ots17‐MH3. Ots17‐MH3 had no individuals assigned using haplotype clustering but had 23 samples assigned based on genotype matching. Genotype‐based matching assigned approximately 12% fewer individuals to haplotypes overall than the phased haplotype clustering (Table S2C) but was better able to assign individuals to the Ots17‐MH3 haplogroup. All GT‐seq samples assigned to a Y‐chromosome haplogroup were genetic males based on the Ots‐SEXY‐3‐1 sex identification assay (Table S3).

The majority of male (phenotypic or genetic) Chinook salmon in Alaska were assigned to Y‐chromosome haplogroups with regional variation in assignment rate. Overall, 90% of phenotypic males (93/103) and 87% of genetic males (232/266) were assigned to a haplogroup. Genetic males that were not assigned Y‐chromosome haplotypes were concentrated in Southeast Alaska; 30 of the 40 unassigned males were from the Little Port Walter and Pullen Creek populations. Only 58% of males from Little Port Walter, and a single male from Pullen Creek could be assigned to a haplogroup. These samples had low missing data, suggesting that the low assignment rate is due to low prevalence of these haplogroups in this region. Excluding these two populations results in 98% haplogroup assignment of male Chinook salmon in Alaska. Distribution of Y‐chromosome haplotypes varied regionally: Y‐chromosome haplotype blocks identified in Alaska were present in nearly all genetic males within Alaska but had rare occurrence outside of Alaska (Table 1). In total, eight males assigned to Ots17‐MH1 were found in British Columbia, and 24 males assigned to Ots17‐MH4 were found in British Columbia, Washington, and Idaho.

### 3.3 Size and age at maturity

**FIGURE 4:**
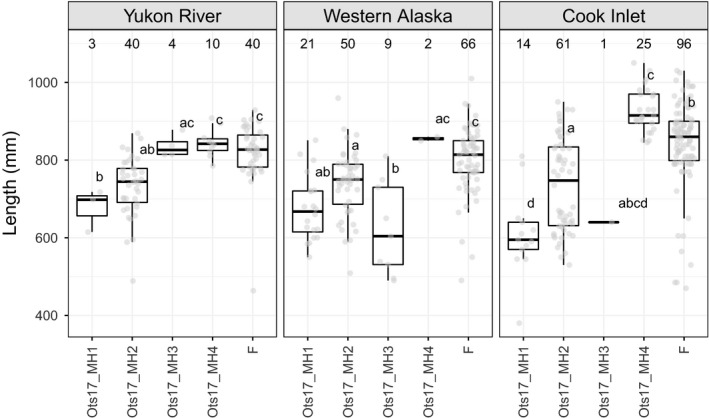
Distribution of size at maturity for each Y‐chromosome haplogroup for Alaska Chinook salmon. Female (F) size at maturity is included for comparison. Samples sizes for each haplogroup are given above the boxplots. Length of each individual is shown by gray points. Within each region, Y‐chromosome haplogroups with statistically different lengths are represented by different letters. Haplogroups with two letters (i.e., ab) do not have statistically different size distributions from haplogroups with a or b

Size‐at‐maturity data were available for nine populations in this study; five populations were genotyped using RADseq, three were genotyped using GT‐seq, and one was genotyped using both RADseq and GT‐seq. Populations were grouped by region (Yukon River, Western Alaska, and Cook Inlet) for visualization. Boxplots of size at maturity for each Y‐chromosome haplogroup showed a consistent relationship throughout these regions (Figure 4). The Ots17‐MH1 haplogroup had the smallest individuals, the OTS17‐MH2 haplogroup was associated with intermediate sized fish, and the Ots17‐MH4 haplogroup was associated with the largest individuals in each region. The Ots17‐MH3 haplogroup showed inconsistent results with small fish in some regions and large fish in others. ANOVA results showed that both haplogroup and population were significantly associated with variation in size at maturity. Post hoc Tukey tests were conducted to determine which haplotypes had statistically significant differences in size. In the Yukon River, individuals with the Ots17‐MH4 haplotype were significantly larger (*p* < .05) than fish with the Ots17‐MH1 or Ots17‐MH2 haplotype. While this pattern was repeated in Western Alaska, the relationship was not statistically significant, likely due to low sample size of the Ots17‐MH4 haplogroup (Figure 4). In Cook Inlet, the Ots17‐MH1, Ots17‐MH2, and Ots17‐MH3 all had significant differences in size.

**FIGURE 5:**
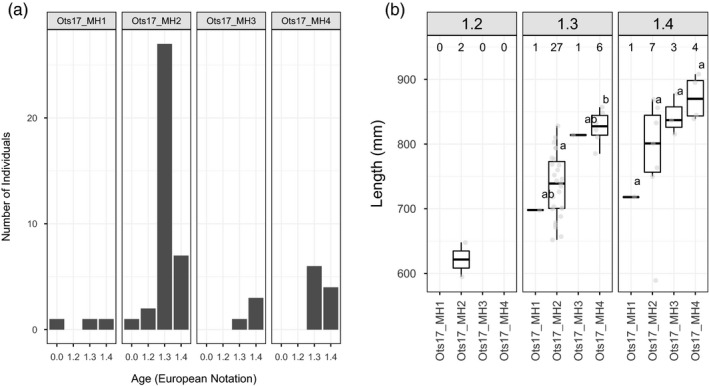
Distribution of (A) age at maturity and (B) size at age for each Y‐chromosome haplogroup in the Yukon River. Length of each individual is shown by gray points. Within each age class, Y‐chromosome haplogroups with statistically different lengths are represented by different letters. Sample sizes for each haplotype are given above the boxplots

Age‐at‐maturity data were available for 177 males from six of the populations in this study (Table 1). The number of males with age data in each population ranged from 19 (Coal Creek) to 57 (Lower Yukon Test Fishery). There was a significant association between Y‐chromosome haplogroup and age at maturity (*n* = 104, *df* = 3, *p* = .047). The distribution of age at maturity by haplogroup was plotted for the Lower Yukon Test Fishery as this collection had the most samples with age data. Histograms of age at maturity for each Y‐chromosome haplotype revealed that the Ots17‐MH2 haplotype had approximately three times as many 1.3 fish as 1.4 fish while the Ots17‐MH4 haplotype had nearly even proportions of 1.3 and 1.4 fish (Figure 5). Boxplots of size at age showed that fish with the Ots17‐MH4 haplotype were larger for age 1.3 and 1.4 than fish with the Ots17‐MH2 haplotype. Results were statistically significant for age 1.3 fish but not age 1.4, possibly due to low sample size. The smallest fish had the Ots17‐MH1 haplotype; however, only two fish in the Lower Yukon Test Fishery had both size and age data for this haplotype.

## 4 DISCUSSION

Variation in life history within populations is common across taxa and is often assumed to be under the influence of many genes of small effect; however, examples of a genes and regions of large effect on life history variation, including single genes, small genomic regions, or chromosome inversions, are increasingly being found (Barson et al., 2015; Johnston et al., 2013; Pearse et al., 2014; Twyford & Friedman, 2015). The genetic mechanism underlying variation in life history has important implications for how life history diversity is maintained under different selective regimes (Hess, Zendt, Matala, & Narum, 2016; Prince et al., 2017), particularly in the case of sexually antagonistic selection where males and females have different phenotypic optima (Barson et al., 2015; Pearse et al., 2019).

Size and age at maturity are ecologically and evolutionarily important traits in Chinook salmon. Numerous studies have examined ongoing declines in age at maturity; however, it has been difficult to disentangle the interactions between environmental and genetic causes of this decline. Size‐ and age‐associated markers and genes have previously been identified in genetic studies of Chinook salmon (Micheletti & Narum, 2018; Waters et al., 2018); however, results were not consistent across populations, and no markers were located on the sex chromosome. We show that a conserved set of Y‐chromosome haplotypes is associated with variation in size and age at maturity in Chinook salmon across the Yukon River, Western Alaska, and Cook Inlet. These observations open a new line of research into the genetic basis of age at maturity in salmonids.

### 4.1 Range‐wide distribution of haplotypes

Chinook salmon are represented by multiple genetically distinct lineages throughout the species range (Beacham et al., 2006; Moran et al., 2012; Waples, Teel, Myers, & Marshall, 2004). These lineages often show little gene flow due to differences in geographic range or spawn timing, and this isolation may result in different sets of Y‐chromosome haplogroups and patterns of recombination across lineages. The Y‐chromosome haplogroups that we identified through extended linkage disequilibrium were consistently observed throughout Chinook salmon populations from the Upper Yukon River south to Little Port Walter in Southeast Alaska. The occurrences become rarer in Southeast Alaska, and very few individuals south of Southeast Alaska could be assigned haplotypes, suggesting that the haplotypes identified within Alaska are regionally restricted. This corresponds to observed breakpoints between Chinook salmon lineages near Cape Fairweather (Templin, Seeb, Jasper, Barclay, & Seeb, 2011), which is approximately 320 km northwest of Little Port Walter and 160 km west of Pullen Creek. While rare, the Ots17‐MH4 haplogroup was found in some individuals as far south as Idaho in the northwestern continental United States, suggesting that Ots17‐MH4 may be broadly distributed even if at low frequency. It is likely that other Y‐chromosome haplogroups exist outside of Alaska, but we were unable to identify them with this dataset. The expanded survey used only a subset of SNPs that characterized the Y‐chromosome haplogroups in Alaska rather than a full RADseq dataset. This approach is useful for surveying previously identified haplotypes but cannot identify new haplotypes that are characterized by other SNPs not included in the GT‐seq panels. The presence of additional haplogroups outside Alaska could be determined by examining reduced‐representation or whole‐genome sequence data from additional populations.

Populations throughout Alaska showed variation in haplotype frequency, which may be ecologically significant given the association between haplotypes and size and age at maturity. Populations from Western Alaska and the Anvik River in the Lower Yukon River had a greater proportion of the Ots17‐MH1 haplogroup, which was associated with smaller fish. Populations in the middle and upper Yukon River and Cook Inlet were primarily composed of Ots17‐MH2 and Ots17‐MH4 haplogroups, which were associated with larger fish. While we did not have adequate samples with size data to characterize size distributions within regions, our finding is consistent with a long‐term analysis of Chinook salmon returns by Lewis et al. (2015). These authors reported smaller fish on average in Kuskokwim and Nushagak River populations from Western Alaska relative to Yukon River and Cook Inlet populations. In addition, the Cook Inlet populations sampled in this study are near and share a common migration pathway with the Kenai River, which has historically produced large Chinook salmon (Lewis et al., 2015; Schoen et al., 2017). The relationship between size and haplotype also varied by region, with fish from the Yukon River having the smallest difference in sizes between haplotypes and fish from Cook Inlet having the largest difference in sizes (Figure 4). While the magnitude of difference appears to be largest in Cook Inlet, it is difficult to accurately assess statistical significance due to the low sample size when splitting samples among regions. Taken together, these results suggest that differing frequencies of Y‐chromosome haplotypes may contribute to regional variation in size of Chinook salmon and that the effect of haplotype on size can vary between regions, potentially due to other genetic influences or different environmental conditions. The Ots17‐MH3 haplogroup was unusual in that it showed no consistent pattern, with large fish in some regions and small fish in other regions (Figure 4). This haplogroup had the smallest sample size, which may affect the trends. This haplogroup also showed the least differentiation from the X‐chromosome based on RADseq data (Figure 3) and may not contain adaptive variants influencing size or age at maturity.

Recombination is generally restricted to the telomeres in male salmon; however, there is evidence that centromeric recombination does occasionally occur (Sutherland, Rico, Audet, & Bernatchez, 2017). The occurrence of rare recombination events could break up haplotype blocks, leading to the degradation of existing haplotypes and the formation of new haplotypes. Recombination events could explain the differences in extended LD observed among the Ots17‐MH‐1, Ots17‐MH‐2, and Ots17‐MH‐3 haplotypes or the variation in extended LD within the Ots17‐MH1 haplotype (Figure 2). However, the variation in extended LD could also represent sequential fixation of alleles due to drift. The boundaries of the haplogroups may also provide clues to the position of *sdY* on chromosome 17. All Y‐chromosome haplogroups overlapped between 0 and ~5 Mb. If the boundaries of the haplogroups are due to rare recombination with the X‐chromosome, then this suggests that the *sdY* gene may be located near this region.

### 4.2 Resolving sexual conflict

Different phenotypic optima for males and females are common across species and can create sexual conflict that is difficult to resolve when adaptive loci are on autosomes. One mechanism is for the same alleles to exhibit sex‐specific dominance, such as the VGLL3 gene that influences age at maturity in Atlantic salmon (Barson et al., 2015). Another mechanism is to partition adaptive variants between nonrecombining regions of sex chromosomes, such as genes governing coloration and fin morphology in Poecilids; these genes are attractive in males but would increase predation risk in females (Lindholm & Breden, 2002). The existence of Y‐chromosome haplotypes demonstrates not only that genetic variation is partitioned between the X‐ and Y‐chromosomes, but that Y‐chromosomes have partitioned different genetic variants (Figure 3). While it is unlikely that the specific SNPs we observed are adaptively important, these haplotypes are associated with variation in adaptively important traits. This suggests that different adaptive variants have been captured by Y‐chromosome haplotypes. Y‐chromosome haplotypes also varied in the chromosomal regions where the X‐ and Y‐chromosomes were differentiated. SNPs that differentiated the Ots17‐MH1, Ots17‐MH2, and Ots17‐MH3 haplogroups from the X‐chromosome were generally concentrated from approximately 4 Mb up to 22 Mb (Figures 2 and 3). One exception was a single SNP at ~600 Kb. SNPs that characterize the Ots17‐MH4 haplogroup were concentrated from ~600 Kb to 6Mb along chromosome 17. The haplogroups may have captured different adaptive variants in each of these regions. This partitioning of genetic variation can resolve sexual conflict in age at maturity and provide a mechanism for the evolution of life history diversity in males. While a lack of recombination between the X and Y chromosome could facilitate adaptive differences between males and females, it is important to note that this would also prevent the purging of deleterious mutations on the Ychromosome, which could result in a complex adaptive landscape.

### 4.3 Importance of Y‐haplotypes for life history diversity

Male Chinook salmon exhibit life history diversity related to maturation age. Older, larger males are believed to have greater reproductive success through their ability to monopolize access to females. Males that mature younger and smaller as jacks are generally believed to have reduced reproductive success but have greater survival to maturation due to reduced risk of ocean mortality (Berejikian et al., 2010). These alternate life histories may exhibit frequency‐dependent fitness, which in theory should exhibit stable proportions; however, this assumes populations are at equilibrium. Male sockeye salmon (*O. nerka*) exhibit similar frequency‐dependent life history variation, but persistent demographic shifts toward an abundance of jacks (males that mature one year earlier than the earliest maturing females) have occurred in some populations as a result of strong selection events coupled with variation in recruitment (DeFilippo et al., 2019). If maturation size and age in males are strongly influenced by an individual's Y‐chromosome haplotype, then size‐selective fishing practices or even size‐selective predation by marine predators (Ohlberger, Schindler, Ward, Walsworth, & Essington, 2019; Seitz, Courtney, Evans, & Manishin, 2019) may result in shifts in haplogroup frequency, demographic changes, and loss of age diversity that are difficult to recover. The markers that we developed can be used to characterize historic (i.e., from archived samples) and current Y‐chromosome haplogroup diversity in Chinook salmon from Alaska to determine whether demographic shifts correspond to shifts in frequencies of Y‐chromosome haplogroups. Ultimately, conservation of life history diversity in Chinook salmon may require conservation of Y‐chromosome haplogroup diversity.

Hypotheses of population structure and delineation of management units using genetic data are typically based on genome‐wide analyses consistent with the assumption that major life history traits are controlled by many genes with small effects. Waples and Lindley (2018) recently commented on the new challenges facing existing conservation frameworks when associations are identified between one or a very few genes and key life history traits. Their comment was prompted by the recent identification of SNPs from a GREB1L gene that explain a large proportion of the variation associated with seasonal timing of adults returning to spawn steelhead (*O. mykiss*) and Chinook salmon (Hess, Zendt, et al., 2016; Prince et al., 2017). Conservation of the Y‐chromosome haplotype shares similar challenges to the GREB1L situation. Waples and Lindley (2018) pose a series of key questions to help provide an informed basis for decisions or management actions. Among other questions, they argue that a full understanding of the distribution of the variation in space and time is needed and that investigations into the genes and mechanisms responsible for the life history variation should be initiated. In the case of the Y‐haplotypes, additional questions exist such as: What are the causal variants within these haplotype blocks? Do haplotype blocks exhibit consistent phenotypes in different environments and with different genetic backgrounds? What proportion of variance in size and age at maturity are explained by these haplotype blocks relative to other regions of the genome? If haplotype blocks are found in other regions of the Chinook salmon range, do they function in a similar manner to those suggested by the results of this study?

## 5 CONCLUSION

Variation in size and age at maturity is common across taxa and is often associated with alternative strategies for increasing fitness; however, the genetic basis of this variation is largely unknown. We identified Y‐chromosome haplogroups that are associated with size, and likely age, at maturity in Chinook salmon throughout Alaska. These haplogroups were primarily restricted to western and southcentral Alaska Chinook salmon where the most diversity in age at maturity exists, and likely represent a subset of the total diversity across the species range. It is possible that each Chinook salmon lineage has a specific set of haplogroups and relationships between haplotypes and size/age at maturity may differ by lineage. Y‐chromosome haplotypes and their potential effect on life history variation in Chinook salmon may provide a basis to help explain the causes and consequences of the recent declines in size and age of adult Chinook salmon, trends that are most pronounced in the region with the highest haplotype diversity. Ongoing efforts to understand the causes of these declines point to size‐specific mortality of maturing fish but also require an unknown evolutionary basis (Ohlberger et al., 2019). Our findings reveal a mechanism for the genetic control of changes in size at age and age at maturity in Chinook salmon. Monitoring haplotype diversity may be particularly important as future changes in environmental conditions and selective fishing may lead to further demographic responses in this economically and ecologically important species.

## CONFLICT OF INTEREST

None declared.

## Supporting information

Fig S1

Table S1

Table S2

Table S3

## Data Availability

Raw GT‐seq data are available in NCBI SRA Bioprojects PRJNA646992 and PRJNA646245.
