# Supplementary figures and images for: Y‐chromosome haplotypes are associated with variation in size and age at maturity in male Chinook salmon
Source: Evol Appl. 2020 Aug 28;13(10):2791–806. doi: 10.1111/eva.13084 (PMC7691470; doi:10.1111/eva.13084)

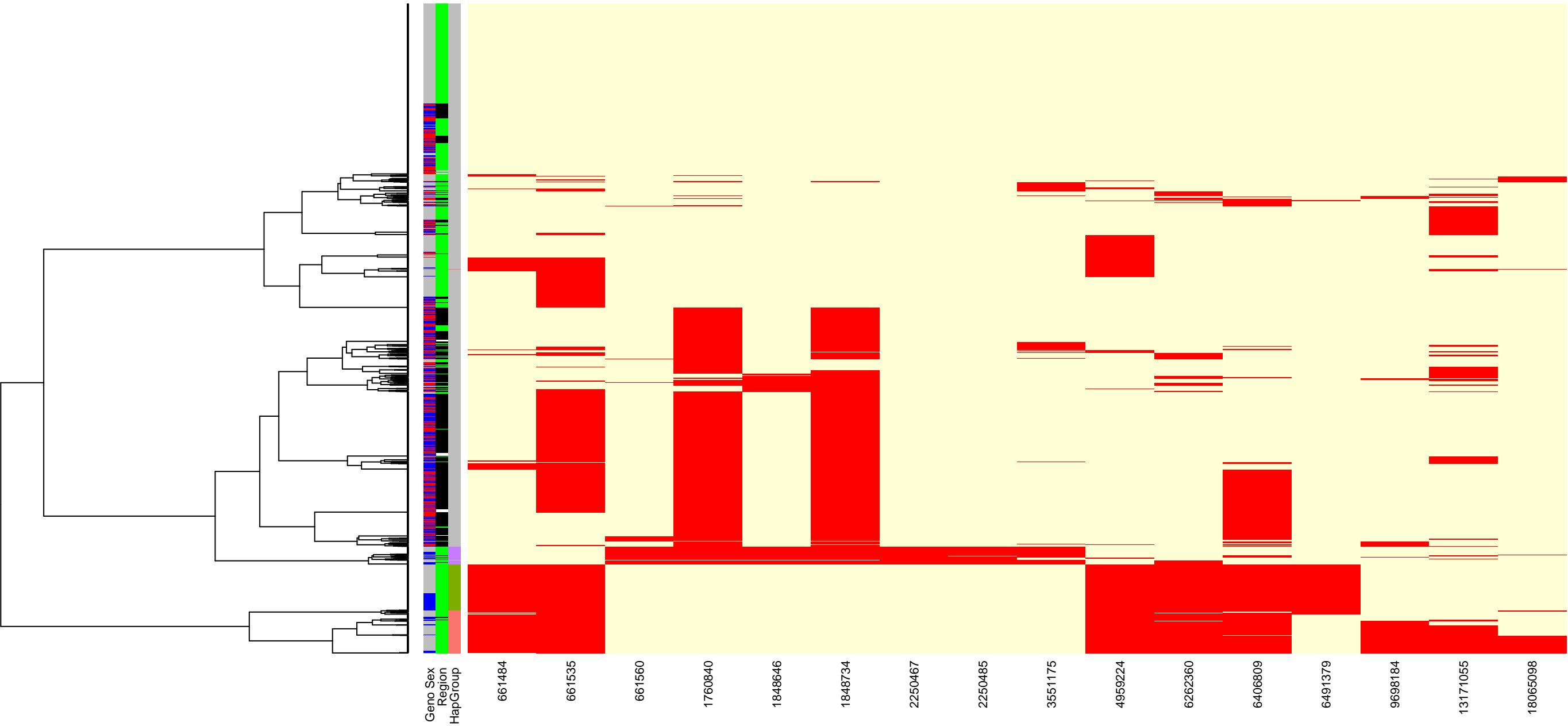

Supplement: Supplementary file 1 — Fig S1 [file EVA-13-2791-s001.pdf]
